# 46 Universal calcinosis in juvenile dermatomyositis: a case report

**DOI:** 10.1093/rheumatology/keac496.042

**Published:** 2022-10-07

**Authors:** N Bahaz, A Remilaoui, F Mechid, H Hafirassou, C Dahou Makhloufi

**Affiliations:** Rheumatology Department, Bab El Oued University Hospital Center, Algiers, Algeria

## Abstract

**Background:**

Dermatomyositis (DM) is an inflammatory myopathy that is often severe and affects children (juvenile DM) in 50% of cases. Subcutaneous calcinosis is seen in 30–70% of juvenile DM and often poses a problem in terms of treatment.

We report a patient with diffuse subcutaneous calcinosis in juvenile DM.

**Observation:**

The patient is K.S, a 17-year-old girl, with background history of congenital heart disease (atrial septal defect), which was corrected surgically in 2021. Since the age of 13, she has had DM for which she has received corticosteroid therapy at a dose of 2 mg/kg/day and improved clinically. The clinical examination reveals subcutaneous nodules in the arms, forearms, phalanx of the right index finger, inner thigh, knees and legs, associated with reduced range of movement of the right elbow (presence of calcifications) and limitation of internal rotation of the right hip.

Laboratory investigations showed no inflammatory syndrome, the muscle enzymes and electromyogram were normal. Radiographs showed calcifications at the sites with subcutaneous nodules.

Bone densitometry showed a decrease in bone mineral density (*Z* score= -3.4 standard deviation at the lumbar spine). The thoracic CT scan showed interstitial lung disease.

The patient was managed with pamidronic acid for the diffuse calcinosis. She is also on diltiazem for Raynaud's phenomenon and hydroxychloroquine.

**Conclusion:**

Subcutaneous calcinosis is diffuse in our patient. Treatment with bisphosphonates (pamidronic acid) is recommended.

